# Genotyping performance evaluation of commercially available HIV-1 drug resistance test

**DOI:** 10.1371/journal.pone.0198246

**Published:** 2018-06-28

**Authors:** Audu Rosemary, Onwuamah Chika, Okpokwu Jonathan, Imade Godwin, Odaibo Georgina, Okwuraiwe Azuka, Musa Zaidat, Chebu Philippe, Ezechi Oliver, Agbaji Oche, Olaleye David, Samuel Jay, Dalhatu Ibrahim, Ahmed Mukhtar, DeVos Joshua, Yang Chunfu, Raizes Elliot, Chaplin Beth, Kanki Phyllis, Idigbe Emmanuel

**Affiliations:** 1 Nigerian Institute of Medical Research, Lagos, Nigeria; 2 Jos University Teaching Hospital, Jos, Nigeria; 3 University College Hospital, Ibadan, Nigeria; 4 AIDS Prevention Initiative in Nigeria, Abuja, Nigeria; 5 Centers for Disease Control and Prevention, Abuja, Nigeria; 6 Division of Global HIV & TB, Center for Global Health, Centers for Disease Control and Prevention, Atlanta, Georgia, United States of America; 7 Department of Immunology & Infectious Diseases, Harvard T.H. Chan School of Public Health, Boston, United States of America; University of Cincinnati College of Medicine, UNITED STATES

## Abstract

**Background:**

ATCC HIV-1 drug resistance test kit was designed to detect HIV-1 drug resistance (HIVDR) mutations in the protease and reverse transcriptase genes for all HIV-1 group M subtypes and circulating recombinant forms. The test has been validated for both plasma and dried blood spot specimen types with viral load (VL) of ≥1000 copies/ml. We performed an in-country assessment on the kit to determine the genotyping sensitivity and its accuracy in detecting HIVDR mutations using plasma samples stored under suboptimal conditions.

**Methods:**

Among 572 samples with VL ≥1000 copies/ml that had been genotyped by ViroSeq assay, 183 were randomly selected, including 85 successful genotyped and 98 unsuccessful genotyped samples. They were tested with ATCC kits following the manufacturer’s instructions. Sequence identity and HIVDR patterns were analysed with Stanford University HIV Drug Resistance HIVdb program.

**Results:**

Of the 183 samples, 127 (69.4%) were successfully genotyped by either method. While ViroSeq system genotyped 85/183 (46.5%) with median VL of 32,971 (IQR: 11,150–96,506) copies/ml, ATCC genotyped 115/183 (62.8%) samples with median VL of 23,068 (IQR: 7,397–86,086) copies/ml. Of the 98 unsuccessful genotyped samples with ViroSeq assay, 42 (42.9%) samples with lower median VL of 13,906 (IQR: 6,122–72,329) copies/ml were successfully genotyped using ATCC. Sequence identity analysis revealed that the sequences generated by both methods were >98% identical and yielded similar HIVDR profiles at individual patient level.

**Conclusion:**

This study confirms that ATCC kit showed greater sensitivity in genotyping plasma samples stored in suboptimal conditions experiencing frequent and prolonged power outage. Thus, it is more sensitive particularly for subtypes A and A/G HIV-1 in resource-limited settings.

## Introduction

The universal access to antiretroviral therapy (ART) for all HIV-infected patients has significantly improved the quality of life of most HIV-infected patients with decreased morbidity and mortality globally. However, detectable viremia occurs in 20–30% of the patients after about 12 months on ART [1]. Incomplete suppression of viral replication could result in the development of HIV drug resistance (HIVDR) mutations which further compromise the efficacy of ART for these patients and may lead to onward transmission of resistant viruses to newly HIV-infected patients. Treatment failure could be caused by either the presence of HIVDR mutations, poor adherence, insufficiently potent drug regimen or decrease in drug level uptake because of poor pharmacokinetic factors [2–4]. Conventional technologies have been used for HIVDR testing using specimens collected from patients suspected to harbour resistant HIV variants. There are two commercially available U.S. FDA-approved genotyping assays, namely ViroSeq and Trugene and several home–brew genotypic assays that have been used or are in use for HIV genotyping [5]. Though the commercially available assays were designed to genotype HIV-1 subtype B virus, they have been used to sequence non-B subtypes with different genotyping sensitivities [6]. They are also expensive and often not affordable for resource-limited settings [7–9] and the production of the Trugene has been discontinued. Thus, there is a need to have access to genotyping kits/assays that are affordable and designed to genotype HIV-1 group M subtypes and circulating recombinant forms (CRFs) that are co-circulating in Nigeria and many West African countries.

The ATCC HIV-1 drug resistance test kit (now being manufactured by Thermo-Fisher Scientific) based on CDC genotyping assay [10] was designed to detect drug resistance mutations (DRMs) in the protease and reverse transcriptase genes of all the HIV-1 group-M subtypes and CRFs. The original assay has been validated for both plasma and dried blood spot (DBS) specimen types with viral load values of ≥1000 copies/ml in Kenya [11] and Uganda [11] and has been used in HIVDR surveys in ART-naïve and–experienced populations including one conducted in Nigeria [12, 13]. However, there has been no independent evaluation for the ATCC HIV-1 Drug Resistance Genotyping Kit manufactured by ATCC (ATCC, Manassas, VA, USA) using samples collected from HIV-1 patients on ART. We performed an assessment on the ATCC kit to determine the genotyping sensitivity using plasma samples stored under suboptimal conditions and its accuracy in detecting DRMs in comparison with ViroSeq assay.

## Materials and methods

### Random selection of stored plasma samples

Between September 2014 and April 2015, 572 stored plasma samples with original viral load (VL) tested at ≥1000 copies/ml and genotyped with ViroSeq HIV-1 Genotyping System 2.0 Assay (Abbott Molecular, Chicago, IL, USA) with successful or unsuccessful genotyping results were retrieved from the sample repository. Among the 572 plasma samples, a sample size of 183 samples was calculated and using a proportionate to size method, 85 ViroSeq successful-genotyped and 98 ViroSeq unsuccessful-genotyped were selected by simple random sampling and used for the current study.

These samples were from patients earlier enrolled for ART at the Nigerian Institute of Medical Research (NIMR), Jos University Teaching Hospital (JUTH), and University College Hospital in Ibadan (UCH) from 2005 to 2010. The samples for this study were collected between 2006–2011 and original VL was measured with Amplicor HIV-1 Monitor version 1.5 (Roche Molecular Diagnostics, Germany) and Cobas Taqman/Cobas Ampliprep 48 and 96 systems (Roche Diagnostics, Branchburg, USA). The samples were then stored at -80°C at the sample repository of these institutions. However, the stored samples experienced frequent and prolonged power outage as experienced in the country. At enrolment, the patients provided informed consent for the use of their samples, approved by the Ethics Committees of Nigerian Institute of Medical Research, Jos University Teaching Hospital, University College Hospital and Harvard T. H. Chan School of Public Health Institutional Review Board. Ethical approval for this study was obtained from the Institutional Review Board of the Nigerian Institute of Medical Research. The participation of CDC investigators with de-identified data was determined as non-human subjects research by the Associate Director for Science at the Center for Global Health, CDC, Atlanta, GA, USA.

### Genotyping using ATCC kits

In 2015, genotyping was performed following the manufacturer’s instructions using the ATCC HIV-1 Drug Resistance Genotyping Kit (ATCC, Manassas, VA, USA) now the kits are manufactured by Thermo-Fisher Scientific [14]. In brief, a 1084 base-pair segment of the 5’ region of the *pol* gene was generated by RT-PCR and nested PCR using the kit Module 1: RT-PCR & Nested PCR (ATCC GK-0098). The purified PCR fragment was then sequenced using the kit Module 2: Cycle Sequencing (ATCC GK-0200), and the sequencing reactions were analyzed on the ABI Prism 3130xl Genetic Analyzer (Applied Biosystems, USA). The customized ReCALL (version 2.25) software was used to edit the raw sequences and generate consensus sequences [15]. Sequence qualities were then confirmed by Stanford HIVDB Calibrated Population Resistance “QA details” to confirm basecalls and eliminate basecalling errors and by the sequence identity matrix analyses using BioEdit. The quality confirmed sequences were analyzed using the HIVdb algorithm, version 8.2 *[https://hivdb.stanford.edu/page/version-updates/]* and HIVDR profiles were compared with the ones from the matched-pair sequences generated by ViroSeq.

### Statistical analysis

The ATCC test results were compared against those from the ViroSeq test on 127 DRMs as identified by mutations obtained using the Stanford HIVdb algorithm version 8.2 and categorized according to the IAS-USA recommendations [16]. Quantitative variables were expressed as median and interquartile range (IQR) unless otherwise stated. Significance in the discordant mutations between the ATCC kit and the ViroSeq assay was assessed using the McNemar test. Analysis of variance was used to test effect of storage duration on genotyping success rate between assays.

### Nucleotide sequence accession numbers

Sequences from this study were submitted to GenBank, and their accession numbers are MF684461 to MF684634.

## Results

### Comparison of genotyping success rate between ViroSeq and ATCC

The 183 samples randomly selected for ATCC assessment had a median VL of 24,270 copies/mL ranging from 2,150–1,746,479 copies/mL. Out of 183, a total of 127 (69.4%) samples were successfully sequenced by either method. Most samples that were PCR amplified, were successfully sequenced. While the ViroSeq system sequenced 85/183 (46.4%) with a median VL of 32,971 (IQR: 11,150–96,506) copies/ml, the ATCC kits successfully genotyped 115/183 (62.8%) samples with a median VL of 23,068 (IQR: 7,397–86,086) copies/ml. A McNemar test of viral load of 47 samples in the lower quartile showed that the two assays were different, p<0.0001 (2 sided). Table 1 shows that both kits successfully genotyped 73 samples with median VL of 33,732 copies/ml but the ATCC kits missed 12/85 (14.1%) samples genotyped by ViroSeq with median VL of 24,783 (IQR: 10,903–105,548). Of the 98 unsuccessful genotyped samples with ViroSeq assay, 42 (42.9%) samples with a median VL of 12,380 (IQR: 5,526–47,333) copies/ml were successfully genotyped using the ATCC kits (Table 1). Both methods were unsuccessful in genotyping 56/183 (30.6%) samples. However, the overall, genotyping rate by ViroSeq assay was 46.4% (85/183) while that of ATCC was 62.8% (115/183), which was a statistically significant difference (p<0.0001).

**Table 1:** Comparing HIV-1 Drug Resistance Genotyping performance between ATCC and ViroSeq methods with different viral load levels.

		ViroSeq genotyped				Total (%) (IQR*), copies/ml
		YES	Median VL^#^ (IQR*), copies/ml	NO	Median VL^#^ (IQR*), copies/ml	
**ATCC genotyped**	Yes	73	33,732 (11,269–96,506)	42	12,380 (5,526–47,333)	115 (62.8) (7,397–86,086)
	No	12	24,783 (10,903–105,548)	56	24,991 (6,595–92,804)	68 (37.2) (7,689.5–92,804)
	Total (%)	85 (46.4)	32,971 (11,150–96,506)	98 (53.6)	13,906 (6,122–72,329)	183

#-VL; viral load

*-IQR: Interquartile range

### Relationship of genotyping rate with duration of sample storage and VL levels

**Fig 1:**
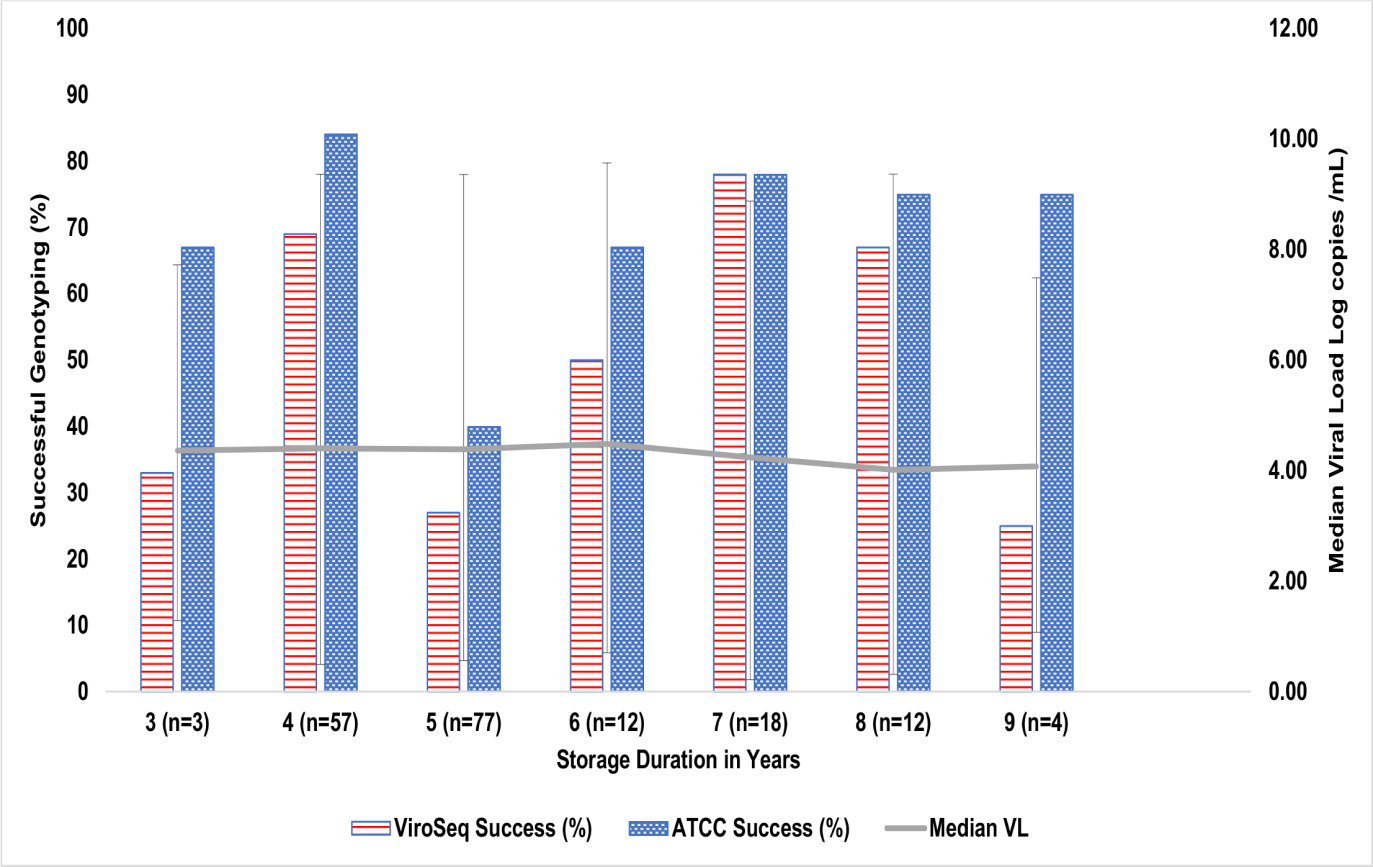
Distribution of genotyped samples by assay used, duration of sample storage and median viral load levels.

The distribution of genotyped samples by the assays used, duration of storage and median VL levels (Fig 1) showed that ≥69.4% of samples stored between three to nine years were successfully sequenced by ATCC assay while the performance of ViroSeq assay was ≤50.0%. The difference in the genotyping performance of both assays for all the samples stored sub-optimally was statistically significant (p<0.05). However, comparing both assays and storage duration did not have any significant effect on their genotyping performance (p>0.05). Though the median VL of samples with longer duration of storage was lower, there was no established relationship between median VL, storage duration and genotyping success rate between the two assays.

### Sequence identity and concordance of detecting drug resistance mutations between the two assays

Sequence identity analysis revealed that the sequences generated by both methods were >98% identical. Among the 73 plasma samples successfully genotyped by both methods, only 29 patients had DRMs. A total of 364 DRMs were found from both assays consisting of 91 minor and 1 major mutations in the protease gene, and 272 mutations in the reverse transcriptase genes. However, in comparison with ViroSeq, table 2 shows that the ATCC kits missed a total of 7 DRMs (2 PIs and 5 NNRTIs) but identified additional 18 DRMs (2 PIs, 8 NRTIs & 8 NNRTIs).

**Table 2:** HIV-1 Drug Resistance mutations against nucleotide reverse transcriptase inhibitors (NRTI), non-nucleotide reverse transcriptase inhibitors (NNRTI) and protease inhibitors (PI) identified by the ATCC assay versus the ViroSeq assay for those individual patients harbouring drug resistance mutations.

SN	PI		NRTI		NNRTI	
	*ATCC	*VSQ	*ATCC	*ViroSeq	*ATCC	*ViroSeq
**1**	L10IV	L10I	**K70EK**, M184IMV	M184MV	Y181C, H221HY	Y181C, H221HY
**2**	K20I	K20I	M184V	M184V	K103N, **H221HY**	K103N
**3**	K20I	K20I	**K219KN**		**Y181CY**	
**4**	L10V, K20I	L10V, K20I	K65R, M184MV, K219EK	K65R, M184V, K219E	K101EK, V108I, Y181C, H221Y	K101E, V108I, Y181C, H221Y
**5**	K20I, L33F	K20I, L33F	M184V, T215Y	M184V, T215Y	K103N, **V108IV**, M230L	K103N, M230L
**6**	K20I	K20I	M184V	M184V	V108I, Y181C, **H221HY**	V108I, Y181C
**7**	K20I	K20I			**K103N, Y181CY**	
**8**	K20I	K20I	M184V, K219EK	M184V, K219E	A98AG, Y181C	A98G, Y181C
**9**	K20I	K20I	M41L, **D67DN**, M184V, **L210LW**, T215Y	M41L, M184V, T215Y	K101E, G190A	K101E, G190A
**10**	K20I	K20I	K65R, Y115FY, M184V	K65R, Y115F, M184V	Y181C, G190A	Y181C, G190A
**11**	L10I, K20I, **L76LQ**	L10I, K20I				
**12**	L10I	L10I	M184V	M184V	V90IV, K103N, Y181C	V90IV, K103N, Y181CY
**13**	**K20I**		D67DN, K70KR, M184V, K219KQ	D67DN, K70KR, M184V, K219KQ	A98G, Y181C	A98G, Y181C
**14**	K20I, K43T	K20I, K43PT	M184I	M184IMV	K103N, V108I	K103N, V108I
**15**	L10I, K20I	L10I, K20I	M184V	M184V	V106M, F227L	V106M, F227FL
**16**	K20I	K20I	D67N, K70E, V75L, Y115F, M184V	D67N, K70E, V75L, Y115F, M184V	K101E, V108I, G190A	K101E, V108I, G190A, **H221HY**
**17**	V11I, K20I	V11I, K20IL	M184V, T215Y, K219KQ	M184V, T215SY, K219KQ	K103N, M230L	K103N, M230L
**18**	K20I	K20I	Y115FY, M184V	Y115FY, M184V	A98AG, Y181C, H221Y	A98G, Y181C, H221Y
**19**	K20I	K20I	**A62AV**, K65R, M184MV	K65R, M184MV	V106A, F227L	V106A, F227L
**20**	K20I	K20I	M184V, K219KQ	M184V, K219KQ	Y181C	**V108IV**, Y181C
**21**	K20I	K20I			V106I, Y181C	V106I, Y181C, **Y188CY**
**22**	K20I	K20I	D67DE, K70EK, M184V, K219KN	D67DE, K70EK, M184V, K219KN	A98AG, V108IV, Y181C	A98G, V108I, Y181C
**23**	K20I	K20I	K70EK, V75I, Y115F, M184V	K70EK, V75I, Y115F, M184V	**V108IV**, Y181C	Y181C
**24**		**L10IL, K20I**	M184IV	M184IV	K101EK, Y181C, G190AG, H221HY	K101EK, Y181C, G190AG, H221HY
**25**	K20I	K20I			K103KN, Y181CY	**A98AG**, K103KN, Y181CY, **H221HY**
**26**	L10I, K20V	L10I, K20V	**A62AV**, K65R, M184IMV	K65KR, M184IMV	K103N, Y181C	K103N, Y181C
**27**	L90M; K20I	L90M; K20I	K65R, Y115F, M184V	K65R, Y115F, M184V	K101E, V108I, Y181C	K101EK, V108I, Y181C
**28**	K20I, E35G	K20I, E35G	**D67DN**, K70EGKR, M184V, **K219EK**	K70EK, M184V	K103N	K103N
**29**	K20I	K20I	M184IM	M184I	**V90IV**, K103NS, Y181C	K103NS, Y181CY

*Despite these differences, both assays yielded 100% similarity in individual patient level HIVDR profile reports. Difference between both methods are in **bold** font types.

^#^Major protease mutation.

### HIV-1 Subtype distribution

The subtype analyses of the 127 samples sequenced showed that 53 (41.7%) samples were subtype G, 48 (37.8%) were CRF02_AG and these were the most common subtypes. Other subtypes identified include CRF06_CPX 7.9% (10), A1 7.1% (9), D 1.6% (2), C 0.8% (1) and some other recombinants which accounted for 3.2% (4). Table 3 shows the distribution of subtypes among samples with ATCC and ViroSeq discordant genotyping success. The table suggests that ATCC may amplify more diverse subtypes but further studies are needed to address this question more directly.

**Table 3:** Distribution of HIV-1 subtypes and recombinants in samples with ATCC and viroseq discordant genotyping successes.

Subtype	*Successfully Genotyped with the assays	
	ATCC successful genotyped & Viroseq unsuccessful genotyped (Frequency)	ATCC unsuccessful genotyped & Viroseq successful genotyped (Frequency)
**G**	14	6
**CRF02_AG**	14	5
**CRF06_CPX**	5	0
**A**	4	0
**C**	1	0
**D**	1	0
**Recombinants (A1/G/K)**	3	1
**Total**	**42**	**12**

* Subtypes were determined on ATCC or Viroseq sequences with successful genotype

## Discussion

HIV-1 drug resistance testing for the purpose of both surveillance and, increasingly for individual patient care is recommended in resource-limited countries, such as Nigeria where treatment has been widely available since 2002. The ViroSeq assay is a U.S. FDA approved test kit designed for HIV-1 subtype B using plasma samples and has been the gold standard assay for HIVDR testing. The plasma samples used for ViroSeq HIVDR testing are required to be stored at -70°C for viability after collection. However, in resource-limited countries, such as Nigeria experiencing frequent and prolonged power outage, maintaining sample integrity at the recommended temperature and conditions has been challenging. This might account for the lower than expected genotyping rate of 46.4% (85/183) obtained during this study by ViroSeq assay. In contrast to ViroSeq assay, the ATCC kit resulted in an overall significantly higher genotyping rate of 62.8% (115/183). For those Viroseq genotyping-negative samples, the ATCC kits were able to genotype 42.9% (42/98) of the samples with lower median VL. The relatively higher genotyping performance of the ATCC kits could have been due to the inclusion of a nested PCR step and the shorter fragment target of 1.1kb as compared to 1.8kb for Viroseq [10]. However, the ATCC kit missed 14.1% (12/85) of the ViroSeq successful-genotyped samples. This may have been because leftover stored RNA extracts were used for ATCC testing for some of the samples analysed while freshly extracted RNA samples were used for Viroseq assay in the current study due to the limited samples available. It was observed that the genotyping performance of both assays was lower than rates reported in a study in Kenya with 94% rates for the ATCC and 78% for ViroSeq) [5]. This finding could be due to frequent and prolonged power outage experienced in the country. During the period of sample storage, often times power outages could last as long as 8hrs/day and occasionally freezers breakdown with downtime of 1-2days before repairs. Samples are left untouched in sites without back-up ultralow freezers. This results in frequent freeze thawing of samples which leads to degradation of the viral nucleic acid resulting in the poor performance of both assays. However, the higher genotyping rate by the ATCC assay across different HIV-1 group-M subtypes and CRFs could be attributed to the fact that the prototype assay for which ATCC kit is based upon had better sensitivity in genotyping diverse HIV-1 subtypes and samples with lower VL than the Viroseq assay [10, 11].

The study findings also raise another very critical and important issue for resource-limited settings, where power outage is a norm, when considering HIVDR testing for the purpose of HIVDR surveillance or individual patient care. The selection of what type of samples to be collected may be the most important decision on the success of the program. Dried blood spot specimens have been extensively evaluated for HIVDR testing in treatment-naïve and -experienced patients [11, 17] and on the transport and storage conditions [10], the World Health Organization is currently recommending that DBS is the alternative sample type for HIVDR surveillance and monitoring purposes if the condition to ensure the quality and integrity of plasma sample cannot be met [18]. Studies have shown that the HIVDR profiles generated from DBS specimens with matched plasma samples are comparable [14, 19]. The prototype assay of the ATCC kits based upon has been extensively used for DBS specimen type and obtained satisfactory genotyping rate [11]. This adds another value for using this assay in resource-limited settings.

Similar to previous studies in the country [20, 21], multiple HIV-1 subtypes and CRFs were found in this study with subtype G and CRF02_AG being the most prevalent. It is important that DR assays used in Nigeria should be robust and able to genotype multiple subtypes and CRFs that are known to co-circulate in the country [21]. Though the ViroSeq kit was designed for subtype B, it was able to genotype all subtypes in this study except for one specimen with subtype C and it also missed out more of the diverse subtypes among the genotyping discordant samples. The ATCC kit, in accordance with its design, genotyped all subtypes in the samples included in this study as also confirmed from other studies [10, 11, 22, 23]. This makes it a suitable kit for use in countries where non-B subtypes are predominant.

The high concordance of the two assays in detecting drug resistance-associated mutations in the plasma samples and the 100% similarity in HIVDR profiles at individual patient level indicates that the ATCC kits can be used for both HIVDR surveillance and routine patient care monitoring. Despite the few minor mutations missed by either assay in both the protease and the RT genes, the clinical interpretation of DR mutations was not affected. More so, it was observed that the cost per test for ATCC kit was half that of Viroseq and reports [10] have shown that using the ATCC assay could reduce the cost of HIVDR testing by 60% thereby making it more affordable for use in resource-limited settings, such as in Nigeria.

In conclusion, this study shows that the ATCC kits have better performance in genotyping diverse strains of HIV-1 group M viruses circulating in Nigeria than the ViroSeq assay. The study also indicates that the ATCC kits had greater genotyping sensitivity in genotyping plasma samples stored under suboptimal conditions. Thus, the ATCC kit is more sensitive particularly for subtypes A and A/G HIV-1 in resource-limited settings where continuous power supply to ensure the integrity of stored samples is challenging.

## Supporting information

S1 File Supporting minimal data. (XLSX)
